# Pain in Children: Neglected, Unaddressed and Mismanaged

**DOI:** 10.4103/0973-1075.76247

**Published:** 2011-01

**Authors:** Lulu Mathews

**Affiliations:** Institute of Palliative Medicine, Calicut, Kerala, India

**Keywords:** Pain in children, Neglected and unaddressed, Barriers to pain assessment and management

## Abstract

Pain is one of the most misunderstood, under diagnosed, and under treated/untreated medical problems, particularly in children. One of the most challenging roles of medical providers serving children is to appropriately assess and treat their pain. New JCAHO regulations regard pain as “the fifth vital sign” and require caregivers to regularly assess and address pain. Pain being a personal experience, many different terms are used to describe different sensations. Assessment of pain in children is linked to their level of development. Children of the same age vary widely in their perception and tolerance of pain.

## INTRODUCTION

Every child will experience pain at one time or another, whether it is from everyday bumps and bruises, or due to more chronic conditions such as headaches, gastrointestinal problems, or diabetes. In fact, as many as 40% of children and adolescents complain of pain that occurs at least once weekly, and chronic pain affects at least 15%–20% of children. Just as chronic pain is more prevalent in women than men, girls report more pain than boys.[1]

Pediatric pain stems from a wide range of chronic conditions- usually muscle, bone, or joint pain, headaches, or abdominal pain-and require pain management. But the medical community has not placed the same emphasis on pain management for pediatric patients as it has for adults and seniors. Each year, 1.5 million children have surgery, and many receive inadequate pain relief and in 20% of cases, the pain becomes chronic. Of children aged 5–17 years, 20% suffer headaches.[2] . More than one-third of children complain of abdominal pain lasting two weeks or longer.[3] Juvenile arthritis, which causes joint inflammation and aches, affects nearly 250,000 people under the age of 16 years.[4]

If left unaddressed, chronic pain can affect children in ways that will follow them throughout their lives. They can develop emotional and psychological scars from their pain that can taint future choices concerning their lives and health care. Untreated pain in childhood also can lead to chronic pain in adulthood and old age[5]

## BE AN ADVOCATE FOR YOUR CHILD’S PAIN MANAGEMENT

Pain is truly both a physical and an emotional experience perceived and processed by the brain, it is a real health problem as well. Each child has different pain perception, and the meaning of pain is also different from child to child. The goal of treatment is to decrease the intensity of pain and make the child feel better. For acute pain, this goal is often met successfully. But chronic pain-pain lasting for at least three months or more-has a different effect on the nervous system and needs to be treated differently. Perhaps one of the most difficult challenges professionally. Perhaps one of the most difficult challenges professionally and emotionally is learning to handle pain in pediatric patients. It is sometimes a necessary part of our work to inflict pain during procedures, immunizations, and other treatments. In the past, there was a relative lack of accountability for providing pain relief. Culture has changed as evidenced by the New JCAHO (Joint Commission on Accreditation of Healthcare Organizations) regulations which regard pain as “the fifth vital sign” and require caregivers to regularly assess and address pain.[6] However, pain remains one of the most misunderstood, under diagnosed, and Under treated/untreated medical problems in children.

## WHY IS PEDIATRIC PAIN NEGLECTED?

Pediatric pain is neglected and undertreated for several reasons:

1. Children and adults react differently when it comes to pain.
2. Doctors focus on the source rather than the symptom. Many physicians become so focused on determining what is causing a child’s pain that they fail to perform pain management.
3. Some physicians do not understand pediatric pain management.

## TELL–TALE SIGNS OF PAIN IN CHILDREN

Certain behaviors can alert you to your child’s pain, even if the child can not properly express it himself. These include:

- Favoring one arm or leg over the other.
- A decrease in physical activity.
- Changes in appetite or sleep pattern.
- Avoiding contact with other children.
- Crankiness, irritability, or unruly behavior.
- Nonverbal expressions of pain such as gasping, wincing, or frowning.
- Physical cues like dull eyes, flushed skin, rapid breathing, or sweating.
- Another way to help your children is to go over the lists of words with them that express pain, so they can use the words that best show what they feel, like “sore,” “itchy,” “burning,” and “aching.”
- Do not rely on just the verbal: Ask children to point to their bodies to show where they hurt and how the pain travels through them.

## PHYSIOLOGY OF PAIN

Pain sensation is a product of several interacting neural systems. Pain transmission can also be modulated by descending pathways that constitute the “analgesia” system. Neonates have the same number of pain nerve endings per square millimeter of skin as adults. They are present in fetus from the second trimester. The central nervous system tracts that subserve pain are completely myelinated by 30 weeks of intra uterine life. Cortical interconnections with the thalamus, those tracts that play a role in higher perception of pain, are complete by 24 weeks. The descending inhibitory controllers of pain, though, are deficient in the neonate controllers of pain, though, are deficient in the neonate. This leads to the likelihood that neonates, particularly preterm neonates, may be more sensitive to pain than older children and adults.[7]

## THE HARM OF PAIN

Pain assists us in avoiding physical harm, but unrelieved pain may be inherently harmful both psychologically and physiologically. Failure to intervene early in children’s pain may lead to impairment in functioning and disruption in families. Unaddressed pain heightens anxiety and fear, which, in turn, increases perception of pain.[8]

## ATTITUDES ABOUT PAIN:BARRIERS TO ADEQUATE PAIN MANAGEMENT IN CHILDREN

We have made a number of advances in our approach to pain management in children, but misconceptions still exist.[9]

1. Many providers believe that children do not remember pain, that children experience less pain than adults, that children are too fragile to receive potent drugs, and that narcotics may induce addiction.
2. Medical caregivers may believe that if one is urgently trying to save a life, there is no time to worry about pain control. Concentration on intensive care may put the neonate into the role of “biologic machine” rather than a human being capable of perceiving, responding to, and interacting with his or her environment.
3. Assumptions on the part of patients as well as caregivers affect pain assessment. Many children will deny pain because of fear of disappointing caregivers or fear of an injection.
4. Many health care providers also at least subconsciously believe that they, rather than the child, can accurately judge a child’s pain experience. They may attribute a child’s distractibility to absence of pain. This perception represents a misunderstanding of the powerful roles of distraction and comforting in the attenuation or relief of pain.
5. We still expect children to react to pain with some predictable, visible signs such as sweating, tachycardia, wincing, crying, jerking away, and muscle tension. The absence of these typical signs may be considered as adaptation on the part of the child.

## CAN’T CHILDREN CATEGORIZE PAIN?

There are several categories of pain. One of the most common is that which is associated with a disease state (e.g., arthritis, sickle-cell disease) or that which is associated with an observable physical injury or trauma (e.g., burns, fractures). Some of the most challenging conditions involve pain that is not associated with a well-defined or specific disease state or physical injury (e.g., tension headaches, recurrent abdominal pain). Pain may also be caused by the medical provider (e.g., circumcisions, injections). Pain may be caused by habits and behaviors as well (e.g., abdominal pain related to intake of alcohol or spicy foods, and so on).

## A DEVELOPMENTAL APPROACH TO ASSESSMENT–NEONATES, TODDLERS, AND SCHOOL GOING KIDS

To accurately assess pain in children, the medical care-giver must tailor assessment strategies to the child’s developmental level. Several factors modify pain perceptions including age, cognition, sex, previous pain experience, temperament, cultural and family factors, and situational factors. There are three widely used categories of behavioral indicators of pain: global rating scales (GRS) behavioral observation scales (BOS). and indirect measures, GRS rely on the assessment of predictable behavioral indicators of pain such as crying, wincing, or screaming. Indirect measures of pain may be assessed by requests for medication, or “well” behavior such as playing. We know, however, that requests for pain medication are not reliably linked to pain intensity. BOS focus on the documentation of specific behaviors indicative of pain. Physiologic measures (e.g., heart rate and blood pressure) are helpful as adjuncts to behavioral observation, but are neither sensitive nor specific pain indicators.

### Neonates

There is no easy or scientific way to tell how much pain an infant is having. BOS and physiologic measures can be difficult to interpret. Neonates may manifest pain by crying or being silent, wiggling, or being still. The infant may make faces or not. The cry of pain in the neonate is quite distinctive. Primary caregivers easily distinguish and interpret cries.

### Toddlers

Reports of caregivers can be invaluable in pain assessment in the toddler age group. The fear factor is a large contributor to experience of pain in this and the school-age group. Toddlers may become very quiet and inactive while in pain or may become very active. Parents report that “they aren’t acting like they normally do.” Interpreting toddlers’ behavior may be difficult due to exacerbating factors such as separation anxiety, memory of previous painful experiences, and physical restraint. Sometimes toddlers manifest their pain and fear by aggressive outbursts.[10]

### School-age children

School-age children are more accurate in communicating about their pain. By age 8 years, children can very reliably describe location of pain. Symptom scales and self-report tools are appropriate for most children 4 years of age and older. Children older than 8 years who understand the concept of order or number can use a Numeric Rating Scale or a Horizontal Word – Graphic Rating Scale. Pain diaries may be helpful in the school age group. School-age children also exhibit self-control when they are experiencing pain. They may not report pain in an attempt to show bravery.[11]

## DISCUSSION

If pain is not addressed and treated early on, it can greatly impact a child’s quality of life by interfering with mood, sleep, appetite, school attendance, academic performance, and participation in sports and other extracurricular activities. Further, if unrelieved, childhood pain can enhance a child’s vulnerability to pain later in life. Early experiences such as pain are associated with multiple alterations in the adult brain in a number of animal models. Repeated exposure to pain may cause altered pain sensitivity, anxiety, stress disorders, hyperactivity, and attention deficit disorder, impaired social skills, and patterns of self-destructive behavior.[8] It is essential that healthcare providers begin to recognize pediatric pain so that appropriate strategies can be devised to target and reduce children’s distress and pain-related disability. Unaddressed pain can also result in significant financial stress for families who not only have to cover healthcare expenses, but who may also have to miss work to care for a sick child. Inadequate prevention and relief of pediatric pain are still widespread. Many obstacles exist to providing appropriate pain care to children and adolescents.

## CONCLUSION

Children are particularly responsive to pain-controlling strategies that involve their imaginations and senses of play. Sensory and procedural information coupled with behavioral techniques can be used to distract children away from painful procedures and to decrease fear and anxiety.

All patients in pain can benefit from well-chosen use of psychologic techniques. This approach in children must take into account the developmental level of the child. Approaches as simple as covering the wound or as involved as play therapy may be used. It is wise to keep children with their caregivers if at all possible. With proper guidance, parents assist with distracting the child and reinforcing the suggestions of the medical team. Developing a calm, patient, understanding approach to the needs of the child and his or her caregivers can markedly enhance the encounter.

In spite of so much understanding about pediatric pain management, the sad reality is that pediatric pain management research has not been effectively translated into routine clinical practice.
